# Editorial: Micronutrients and critically ill patients

**DOI:** 10.3389/fmed.2023.1352808

**Published:** 2024-01-08

**Authors:** Taline Lazzarin, Paula Schmidt Azevedo, Barbara Rita Cardoso, Vivian Marques Miguel Suen, Marcos Ferreira Minicucci

**Affiliations:** ^1^Department of Internal Medicine, Botucatu Medical School, São Paulo State University - UNESP, Botucatu, Brazil; ^2^Department of Nutrition, Dietetics and Food, Monash University, Melbourne, VIC, Australia; ^3^Victorian Heart Institute, Monash University, Clayton, VIC, Australia; ^4^Department of Internal Medicine, University of São Paulo, Ribeirao Preto, Brazil

**Keywords:** micronutrients, critically ill patients, mortality, vitamins, electrolyte

Vitamins, trace elements, and electrolytes are known as micronutrients and play a crucial role in metabolism and the maintenance of tissue function. In the intensive care unit (ICU) context, there is a risk of micronutrient deficiency due to several factors, such as inadequate dietary intake, increased requirements, acute renal injury, and low exposure to sunlight. On the other hand, the inflammatory state observed in these patients reduces the serum levels of many micronutrients without a true deficiency (1, 2).

The current main guidelines suggest that micronutrient deficiency negatively impacts health outcomes, such as mortality and quality of life. Thus, the prevention of nutritional deficiency must be prioritized by ensuring the daily requirements are met through enteral or parenteral nutrition, as well as monitoring and treating known deficiencies. However, the benefits of supraphysiological supplementation need to be clarified, and there is an urgent requirement for more evidence-based studies to address which and how much micronutrients should be supplemented to patients in intensive care. Therefore, this Research Topic aimed to publish articles examining micronutrient status or the effects of micronutrient supplementation in critically ill patients (3–5). Four articles were published as part of this collection, bringing some perspectives to help understand the role of micronutrients in critically ill patients.

Hill et al. published an update on the effects of vitamins D and C in critical illnesses. This insightful narrative Review explores these micronutrients in terms of pathophysiology, benefits, potential risks, and guideline recommendations. The Review addresses the role of vitamin D in calcium homeostasis, endothelial function, and the immune system. Additionally, it summarizes practical applications observed in trials using vitamin D in the critical care setting. An important article cited by Hill et al. was the VITDAL trial (6), which showed lower mortality with vitamin D supplementation in patients with severe vitamin D deficiency (vitamin D < 12 ng/ml). It is crucial to note that this was a subgroup analysis, and many studies have failed to show robust benefits of indiscriminate vitamin D supplementation to critically ill patients. This underscores the idea that supplementation should be reserved for patients with proven and clinically evident nutritional deficiencies. Regarding vitamin C, the authors emphasize the importance of this micronutrient for immune, coagulation, cardiovascular, respiratory, central nervous system, and renal functions. Additionally, they discuss the different subgroups in ICUs, such as patients in the acute phase of critical illnesses, that may benefit from vitamin C administration due to the attenuation of oxidative stress and systemic inflammation. Furthermore, the authors address ongoing research scenarios involving vitamin C supplementation to patients with acute respiratory distress syndrome (ARDS), a history of cardiac surgery, cardiac arrest, and severe burns.

The role of vitamin C was also addressed in COVID-19 patients in this Research Topic. Vitamin C is known for its antioxidant capacity and contributes to immune function, which may be helpful during infectious episodes. During the COVID-19 pandemic, it was proposed that vitamin C supplementation could be beneficial for disease recovery. In this context, Gavrielatou et al. conducted a retrospective analysis of adult patients with ARDS due to COVID-19 who underwent mechanical ventilation in a university ICU in Greece. In this study, 113 patients met the inclusion criteria, of which 10 received high doses of vitamin C concomitantly with thiamine. The role of thiamine as an immunomodulator in the response to COVID-19 is not yet well elucidated, but the results of this study could have been influenced by thiamine supplementation. In this cohort, no significant differences were found in the evaluated outcomes of mortality, days of ICU admission, days of mechanical ventilator use, dialytic support, and vasopressor use. In support of this finding, the same group conducted a Systematic Review and meta-analysis on the subject. Eleven studies with 1,807 COVID-19 patients were included, including six observational studies and five randomized controlled trials (RCTs). In the meta-analysis of this review, there was no difference in mortality between the group that received vitamin C and the control group (standard-of-care). It is noteworthy that sample heterogeneity was significant (I^2^ = 74%), but the absence of association was maintained even when a sensitivity analysis was performed with low-risk bias studies or by selecting only RCTs. Despite the null effects of vitamin C supplementation, some recent evidence suggests that the administration of vitamin C combined with other antioxidant substances, such as quercetin, has promising results in the negative conversion of SARS-CoV-2 RT-PCR tests, resolution of acute symptoms, and modulation of the inflammatory response in mild to moderate COVID-19. However, more studies in critically ill patients are necessary to recommend vitamin C supplementation (7).

With this in mind, Zhao et al. published an RCT protocol that will be applied in China to investigate whether vitamin C supplementation at high doses can be beneficial to patients with sepsis. The authors propose including 620 patients with sepsis who will receive vitamin C (200 mg/kg/24 h) or a placebo for 4 days. The primary outcome is mortality at 28 days, and secondary outcomes are the incidence of organ failure and adverse events.

Although vitamins and trace elements are the most studied micronutrients in critically ill contexts, we cannot underestimate the importance of electrolytes. Owing to the high prevalence of phosphorus disorders in critical illness, Zheng et al. discussed the role of hyperphosphatemia as a prognostic biomarker in critically ill patients. The authors conducted a systematic review and meta-analysis that included 10 articles, encompassing over 60,000 patients in ICUs, trauma centers, or large burn units. The findings revealed that hyperphosphatemia was associated with an increased risk of mortality (OR 2.82, 95% CI 2.35–3.38, random-effects model). Such an association remained significant after adjustment for confounders, such as sample size, geographical location, the use of an initial serum phosphate level, measurement unit, duration of follow-up, or mortality prevalence. The authors indicated that there is a greater need for renal replacement therapy in hyperphosphatemic patients.

The studies published in this Research Topic bring new perspectives to enhance the understanding of the role of micronutrients in critically ill patients. However, the literature still has significant gaps that urgently need to be addressed. It is crucial to elucidate several aspects related to supplementation trials, such as supplementation doses, administration route, timing, duration of intervention, and the selection of endpoints. Clinical trials commonly focus on single micronutrients, considering the treatment from a “drug” perspective. However, emerging research suggests that administering a combination of micronutrients may be more effective because the antioxidant defense is an intricate and complex system that does not depend on a single micronutrient but on the interplay between various substrates. Consequently, forthcoming trials emphasize that the early multi-micronutrient approach might bring the benefits that single-micronutrient supplementation failed to (8, 9).

## Author contributions

TL: Conceptualization, Methodology, Writing—original draft. PA: Supervision, Validation, Writing—review & editing. BC: Conceptualization, Supervision, Writing—review & editing. VS: Conceptualization, Supervision, Writing—review & editing. MM: Conceptualization, Supervision, Validation, Writing—review & editing.
